# Unsatisfactory long-term virological suppression in human immunodeficiency virus-infected children in the Amazonas State, Brazil

**DOI:** 10.1590/0037-8682-0333-2020

**Published:** 2020-10-21

**Authors:** Ana Luisa Opromolla Pacheco, Meritxell Sabidó, Wuelton Marcelo Monteiro, Solange Dourado de Andrade

**Affiliations:** 1 Universidade do Estado do Amazonas, Departamento de Medicina, Manaus, AM, Brasil.; 2 Fundação de Medicina Tropical Dr. Heitor Vieira Dourado, Programa de Pós-Graduação em Medicina Tropical, Manaus AM, Brasil.; 3 Universitat de Girona, Department of Medical Sciences, Catalunya, Spain.

**Keywords:** Highly active antiretroviral therapy, Child, Adolescent, HIV, Sustained virologic response

## Abstract

**INTRODUCTION:**

Achieving viral suppression (VS) in children is challenging despite the exponential increase in access to antiretroviral therapy (ART). We evaluated VS in children >1 year of age and adolescents 5 years after they had begun ART, in Manaus, Amazonas state, Brazil.

**METHODS:**

HIV-infected**,** ART-naive children >1 year of age between 1999 and 2016 were eligible. Analysis was stratified by age at ART initiation: 1-5 y, >5-10 y, and >10-19 y. CD4^+^ T-cell count and viral load were assessed on arrival at the clinic, on ART initiation, and at 6 months, 1 year, 2 years, and 5 years after ART initiation. The primary outcome was a viral load <50 copies/mL 5 years after ART initiation.

**RESULTS:**

Ultimately, 121 patients were included. The mean age at diagnosis was 4.8 years (SD 3.5), mean CD4% was 17.9 (SD 9.8), and mean viral load was 4.6 log10 copies/ml (SD 0.8). Five years after ART initiation, the overall VS rate was 46.9%. VS by patient age group was as follows: 36.6% for 1-5 y, 53.3% for >5-10 y, and 30% for >10-19 y. Almost all children (90,4%) showed an increase in CD4%+ T cell count. There were no statistically significant predictors for detecting children who do not achieve VS with treatment. VS remained below 65% in all the evaluated periods.

**CONCLUSIONS:**

Considerable immunological improvement is seen in children after ART initiation. Further efforts are needed to maintain adequate long-term VS levels and improve the survival of this vulnerable population.

## INTRODUCTION

The rate of mother-to-child transmission of human immunodeficiency virus (HIV) in low-and-middle-income countries (LMIC) has been reduced by more than half since 2000, from about 37% to 15% in 2014^1^. However, in the same year, only 50% of all HIV-exposed infants had been tested before reaching their second month of life^2^. For infants who are tested, delays in obtaining results and further losses in the testing-to-treatment cascade still occur; therefore, only 30% of perinatally-infected children are effectively linked to clinics and started on antiretroviral therapy (ART) in a timely manner. As a result, for many children and adolescents, an HIV diagnosis is made in late childhood after many years of ill health^3^ ^-^ ^5^.

The introduction of highly-active ART led to a significant reduction in mortality and an increase in the quality of life of people affected by the disease^1^ ^,^ ^2^. Since 2010, many improvements in access to ART for people living with HIV have been observed; by 2015, global ART coverage had more than doubled^6^. However, to end the Acquired Immunodeficiency Syndrome (AIDS) epidemic, provision of universal health coverage is insufficient. The commitments for the next five years include efforts to accelerate and focus on HIV prevention, efforts to enable people to discover their HIV status, as well as providing ART and comprehensive long-term care to all people living with HIV^7^.

Nevertheless, children constitute a population for which it is difficult to achieve these goals^8^. Achieving VS in the pediatric population is challenging for many reasons, the most common being variation in weight gain, pharmacokinetics of ART, drug resistance due to prior exposure to interventions from mother-to-child vertical transmission of effects, and adherence problems due to poor palatability of drugs or dependence on caregivers^9^.

Several studies have reported VS rates in children on ART, but global summary estimates of long-term outcomes are lacking^7^. In addition, previous studies did not cover factors that underpin the pediatric ART response. In Brazil, by the end of 2016, there were approximately 830,000 cases of HIV infection and AIDS. Of those, 54% achieved VS^10^. However, there are no specific data on the cascade and care continuum for Brazilian children^10^.

Our aim was to evaluate VS in children and adolescents who started therapy late in infancy (after the age of 12 months) up to 5 years after the initiation of ART.

## METHODS

### Study site

A cohort study was conducted by the HIV/AIDS pediatric clinic at Fundação de Medicina Tropical Doutor Heitor Vieira Dourado in the city of Manaus, Brazil. This teaching hospital is an ART referral center for children living with HIV. It provides HIV care within the public health system and treats the highest number of HIV patients in the state of Amazonas.

### Study population

All children and adolescents who visited the HIV/AIDS pediatric center between 1999 and 2016 were eligible. The patients were included if they had a confirmed HIV diagnosis, were treatment-naïve (the mother received no treatment or prophylaxis during pregnancy or childbirth), were started on ART after 12 months of age at our center, and were alive after 5 years of continued ART. They were excluded if they visited the center only once. Patients were considered HIV-positive if they showed positive results on at least one of the following, taken at different times: 1) a test for HIV antibodies by enzyme-linked immunosorbent assay and western blot carried out at the age of 18 months or older, or 2) two plasma quantitative viral RNA test results above the detection level in separate blood specimens obtained at the age ≥2 months old (until 2008) and at 1 month old (after 2008). They were assessed from the date of ART initiation until 5 years after ART initiation or until they were lost to follow-up or died, whichever occurred first.

### Data collection

The patients’ electronic medical records were reviewed in order to retrieve demographic information, which included gender, place of birth, age at first visit, HIV diagnosis and ART initiation, time between first visit and ART initiation, year of ART initiation in or after 2009, born at term (No/Yes), primary caregiver (parents, others), orphan (mother/father/both absent), transmission route, and the number of hospital admissions prior to HIV diagnosis as specified by their caregivers (a proxy for morbidity that was defined as frequency of hospital admissions prior to HIV diagnosis). The clinical and immunological staging after ART initiation was defined according to the Center for Disease Control and Prevention (CDC)’s revised classification of 2008 for children <13 and 13-18 years old^11^and categorized as mild, moderate, and severe immune suppression. CD4^+^ T-cell count at ART initiation results was expressed as absolute values and percentage and the viral load (VL) was expressed as log/mL at the time of ART initiation.

The following information was collected: mother’s age at the start of pregnancy and obstetric data, such as prenatal care received, mode of delivery, breastfeeding and duration. Any medical consultation that the mothers received during gestation was considered prenatal care. Maternal breastfeeding at any time and for any duration was defined as maternal breastfeeding.

The details of the first ART received, as per national guidelines, were recorded. Before 2009, the decision to start ART was based on clinical staging or CD4^+^ T-cell count, and the goal was not to achieve VS, but reduction in VL and improvement in clinical and immunological outcomes^12^ ^,^ ^13^. The guidelines were revised in 2009, when ART was recommended for all children <12 months regardless of CD4^+^ T-cell count^14^, and, again in 2017, when the recommendation was expanded to universal treatment^15^. Since 2009, the primary goal of ART has been to achieve VS.

Regarding first-line regimens, until 2006, there were three options according to clinical and immunological status: two nucleoside reverse transcriptase inhibitors (NRTIs), three NRTIs, or two NRTIs plus one non-nucleoside reverse transcriptase inhibitor (NNRTI)/protease inhibitor (PI). From 2007 onwards, two NRTI, plus one NNRTI/IP was recommended. The consultations, medications, and exams were free of charge and provided by the Brazilian Public Health System (*Sistema Único de Saúde* - SUS) for all patients.

The data were collected at six time points: arrival at the pediatric HIV center, immediately before the initiation of ART; 6 months after ART; and 1, 2, and 5 years after ART initiation. Clinical and biological monitoring procedures were carried out as per the national guidelines: clinical assessment was scheduled every month or 2 months, and immunological (CD4^+^ T-cell count) and virological monitoring (VL-measurement), every 6 months.

VL was quantitatively assessed by b-DNA (Versant HIV-1 RNA 3.0 Assay, Bayer, Chiron Diagnostics, Emeryville, CA, USA) until 2013 (detection limit of 50 copies/mL), and by Abbott Real Time HIV-1 assay (Abbott Laboratories, Abbott Park, Illinois, USA), after 2013 (with a detection limit of 40 copies/mL), and the results were expressed in log10 HIV-1 RNA copies/mL. Until 2008, there was only an absolute number of CD4^+^ T-cell counts available. Thereafter, the results were recorded in absolute numbers and percentages. The CD4^+^ T-cell counts and VL were recorded closest to the six time points related to ART initiation, with a window of up to ±6 months. All data were entered into a database by a pediatrician.

### Study Outcome

The primary outcome was VS defined by VL <50 copies/mL among those who were alive and on treatment 5 years after ART initiation. The analysis was stratified by age at ART initiation, since these groups can present different behavior and immunological responses. Groups were defined as follows: young children (1 to 4.9 years), older children (5 to 9.9 years), and adolescents (according to WHO definition, ≥10 years). We also compared VS levels before and after 2009, since from 2009 onwards, the primary goal of ART was to achieve VS, as previously mentioned.

The optimal virological response was defined as an HIV VL of <50 copies/mL in >90% of the samples, while suboptimal response and poor responses implied an HIV VL of <50 copies/mL in 50-90% and <50% of the samples, respectively (definitions according to Guillen et al., 2007)^16^.

### Statistical analysis

Descriptive statistics of patient characteristics at baseline and during follow-up were performed using patient age group. Continuous variables were summarized using mean and standard deviation (SD); categorical variables were summarized using frequency and proportions. Differences between age groups were compared using chi-square statistics for categorical variables and ANOVA for continuous variables. A *t*-test was used to evaluate the evolution of CD4^+^ T-cell count at different time points. Bivariate methods were used to examine the relationships between independent variables and the primary outcome of VS at 5 years after treatment initiation. We performed a multivariate logistic regression analysis using the enter method (all variables entered at the same time regardless their significance level in bivariate analysis), to identify independent predictors of virological suppression at year 5. Statistical analyses were performed using the SPSS software package, IBM SPSS Statistics for Windows, version 11.5 (IBM Corp., Armonk, N.Y., USA). The significance level was set at 0.05. 

### Ethics

The study protocol was approved by the Ethical Review Board at FMT-HVD (number 36157214.3.0000.0005). The procedures followed were in accordance with the ethical standards of the responsible committee on human experimentation and in accordance with the principles of Declaration of Helsinki, 1964, as revised in 1975, 1983, 1989, 1996, and 2000.

## RESULTS

### Patients characteristics

**FIGURE 1:**
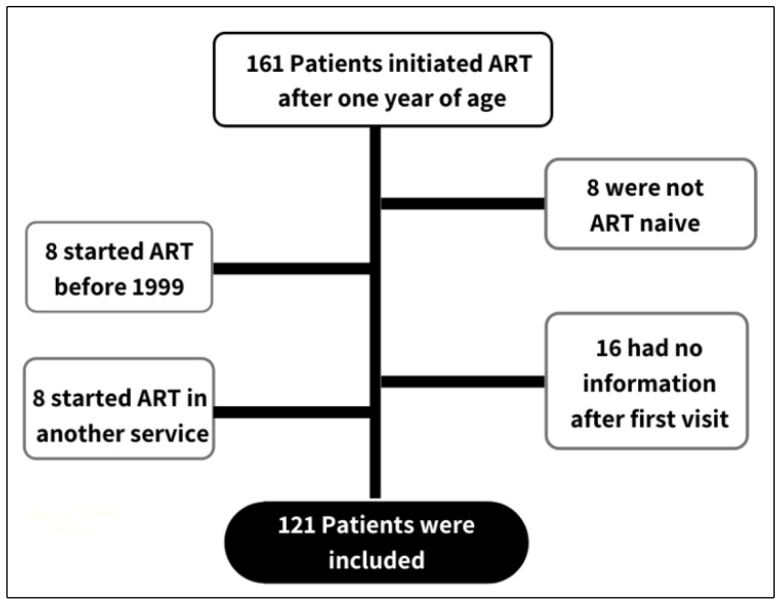
Flowchart of cohort enrollment for analysis of viral suppression after 5 years of first-line ART.

From 1999 to 2016, 161 ART-naïve infected children were initiated on ART. A total of 121 HIV-infected children and adolescents fulfilled the inclusion criteria and were subsequently analyzed (Figure 1). 

The patients were mainly from Manaus (83.5%), and 52.9% were female. The parents were the primary caregivers of 63.6% of the patients, and 35% were orphans (mother/father/both absent). The transmission route was vertical in 96.0% of the patients. The mean age on the first hospital visit was 4.8 years (SD, 3.5). Less than half of the patients (41.3%) who arrived at our clinic were between the ages of 5 and 19 years, which indicates that they were older than 5 years when they were diagnosed. All patients were born to mothers who were unaware of their HIV status during pregnancy, labor, or breastfeeding and therefore did not receive timely treatment or prophylaxis. Before their HIV diagnosis and before arriving at our service, all of our analyzed patients were hospitalized for various reasons at least once (mean number of hospitalizations, 1.1; SD, 1.2). On HIV diagnosis, up to 68.6% had moderate or severe immunodeficiency, according to the CDC classification. 

The mean age for ART initiation was 6.3 years (SD, 4.1). Almost half (48.7%) started ART in or after 2009, and for 60.3%, the preferred initial regimen was 2 NRTI + 1 NNRTI. Their mothers had a mean age of 23.8 years (SD, 5.5) at the start of pregnancy, 66.9% had received some form of prenatal care, none of the patients were exposed to maternal ART during pregnancy or delivery, 59% were born by vaginal delivery, and 78.5% of the children were breastfed with a mean breastfeeding duration of 17.7 months (SD, 15.9). 

### Long-term immunological and virological response to antiretroviral therapy

**FIGURE 2:**
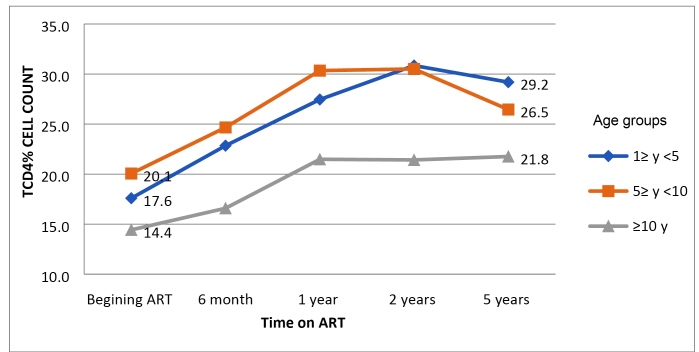
Proportion of CD4^+^ T-cell count recovery on ART according to age during antiretroviral therapy. *Note:* Recovery after 5 y. TCD4%: p=0.00001 for children 1≥ year ˂5; p=0.00001 for children 5≥ years ˂10; and p=0.21 for children ≥10 years.

Eighty-three (68,5%) children had moderate-to-severe immune deficiency at the time of ART initiation: young children (1-5 y) and older children (>5-10 y) had higher CD4^+^ T-cell count %, 18.3% and 19.1%, respectively, than adolescents (>10-19 y), 14.4%. The CD4^+^ T-cell count % increased gradually overall, but a significant increase was only seen up to 10 years of age (P <0.004) after 5 years on ART (Figure 2). There was no difference in the CD4^+^ counts over time between VS patients and non-VS patients.

At the time of ART initiation, young children had a higher HIV VL than older children and adolescents (Table 1). During the 5 years of treatment, a significant decline in HIV VL was observed in all three age groups: 2.26 (SD, 1.95), 1.56 (SD, 1.67), 3.03 (SD, 2.17) (P < 0.001). 

**TABLE 1:** Characteristics of 121 HIV-infected children and adolescents in Manaus (Brazil).

Characteristics	Total	Young Children	Older Children	Adolescent
	n = 121	(1≥ years <5)	(5≥years <10)	(≥10 years)
		n = 71	n = 30	n = 20
Gender - male (%)	57 (47.1)	35 (49.3)	9 (30.0)	13 (65.0)
Place of birth				
Manaus (%)	101 (83.5)	57 (80.2)	27 (90.0)	17 (85.0)
Other (%)	19 (15.7)	13 (18.3)	3 (10.0)	3 (15.0)
Parents as primary caregivers	77 (63.6)	48 (67.6)	15 (50.0)	14 (70.0)
Orphan - yes (%)	44 (36.3)	24 (39.3)	11 (37.9)	9 (60.0)
Vertical transmission (%)	116 (96.0)	70 (98.6)	30 (100)	19 (95.0)
Born at term (%)	98 (81.3)	53 (74.6)	27 (90)	19 (95)
Age at first visit in years, mean (SD)	4.8 (3.5)	2.5 (1.3)	5.6 (1.9)	9.0 (4.1)
Age at HIV diagnosis in years, mean (SD)	4.8 (3.5)	2.5 (1.3)	5.6 (1.9)	9.0 (4.1)
Age at ART initiation in years, mean (SD)	6.3 (4.1)	3.0 (1.7)	7.6 (3.7)	12.3 (5.6)
Time between first visit and ART initiation in months, mean (SD)	19 (30)	7 (10)	22 (22)	42 (48)
ART initiation ≥ 2009	59 (48.7)	34 (49.6)	18 (61.6)	10 (50.0%)
Number of hospitalizations, mean (SD)	1.1 (SD)	1.3 (1.4)	1.0 (1.2)	1.0 (1.3)
Clinical stage at ART initiation				
A	33 (27.3)	22 (30.0)	13 (44.0)	8 (40.0)
B	73 (60.3)	42 (60.0)	15 (50.0)	9 (45.0)
C	10 (8.3)	7 (10.0)	2 (6.0)	2 (8 .0)
VL (log/ml) at ART initiation, mean (SD)	4.6 (0.8)	5.0 (0.8)	4.3 (0.8)	4.4 (0.6)
LT-CD4 at ART initiation				
Absolute number, mean (SD)	555.7 (433)	669.2 (424)	563 (363)	257(198)
%, mean (SD)	17.9 (9.8)	17.6 (8.9)	20 (9.3)	14.4 (9.3)
First ART				
2NRTI	16 (13.2)	12 (17.0)	4 (13.3)	0
2NRTI + 1NNRTI	73 (60.3)	30 (42.2)	18 (62.2)	14 (70.0)
2NRTI + 1PI	32 (26.4)	29 (40.8)	8 (26.6)	6 (30.0)
**Pregnancy characteristics**				
Mother age at the start of pregnancy, mean (SD)	23.8 (5.5)	22 (5.0)	24 (4.0)	22 (5.0)
Prenatal care received (%)	81 (66.9)	44 (61.9)	23 (76.6)	14 (70.0)
Vaginally delivered (%)	71 (59.0)	37 (52.1)	22 (73.3)	10 (50)
Breastfeeding - Yes (%)	95 (78.5)	49 (69)	29 (96.6)	17 (85)
Duration of breastfeeding in months, mean (SD)	17.7 (15.9)			

*Note:* Data is number and proportion unless otherwise indicated.

**FIGURE 3:**
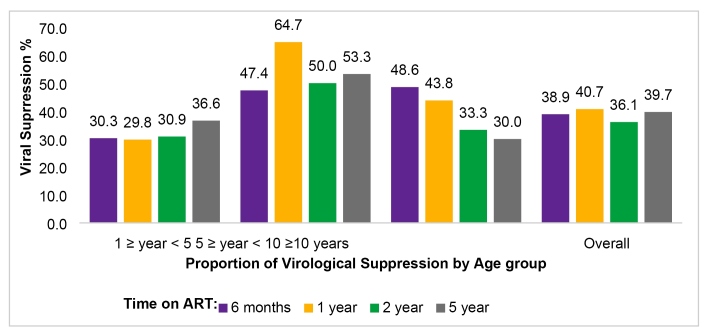
Virological suppression according to age group at different time points after ART.

This study also aimed to determine whether social, demographic or clinical characteristics could predict viral suppression. All children had a higher viral load in the pre-ART period and showed a good clinical response after initiation of ART. In the groups of older children and adolescents, an increase in the percentage of children with VS was found after one year of ART, whereas in the group of young children, the percentage stabilized at around 30% across the five years of follow-up treatment. Five years after ART initiation, the proportion of patients with VS by age group was 37.1% in young children, 55.7% in older children, and 30.0% in the adolescent group. The proportion of patients who achieved undetectable VL decreased in accordance with the duration of ART (Figure 3). When we analyzed VS and year of treatment initiation, the patients who started ART after 2009 had a higher rate of VS (P = 0.043) than those who started it before 2009. 

In bivariate analysis, gestational age showed a trend towards positive correlation with VS, however in multivariate analysis, there were no identifiable predictors to detect children who will develop VS during treatment (Table 2).

**TABLE 2:** Bivariate and Multivariate analyses for VS <50 cp/mL after 5 years of ART.

Variable*	Bivariate analysis		Multivariate analysis	
	Odds ratio (95% CI)	p value	Odds ratio (95% CI)	p value
Gender (M/F)	1.02 (0.45-2.29)	0,94	0.74 (0.20-2.64)	0.64
Orphan (No/Yes)	1.06 (0.35-3.15)	0.40	1.42 (0.29-6.89)	0.65
Caregivers (parents/others)	1.32 (0.55-3.13)	0.53	0.67 (0.16-2.78)	0.58
Born at term (No/Yes)	2.17 (1.70-2.78)	0.06	**	
Place of birth (other/Manaus)	0.88 (0.24-3.16)	0.20	2.90 (0.31-3.65)	0.34
Breastfed (No/Yes)	1.33 (0.27-6.40)	0.71	0.42 (0.01-10.9)	0.60
Age at HIV diagnosis (<5 y/≥5 y)	0.83 (0.37-1.86)	0,20	0.34 (0.42-2.76)	0.31
Age at time of ART initiation (<5 y/≥5 y)	0.99 (0.44-2.21)	0,27	2.68 (0.26-3.77)	0.40
Time between arrival and ART initiation (<6 mo/≥6 mo)	1.09 (0.49-2.45)	0,79	1.97 (0.42-9.20)	0.38
CDC at ART initiation (N+A/B+C)	0.63 (0.26-1.51)	0.70	3.24 (0.70-15.01)	0.13
HAART therapy (INTRNN/IP)	0.88 (0.37-2.13)	0.79	0.64 (0.15-2.65)	0.53
Pre-natal care received (No/Yes)	0.44 (0.07-2.59)	0.60	2.51 (0.16-3.86)	0.51
Mother’s age at birth (≥20 y/<20 y)	1.07 (0.47-2.66)	0.79	0.32 (0.07-1.42)	0.13

*Note: during data collection, a window of ±6 months was stipulated to accept the viral load values. In 25 patients, the dates were greater than 6 months, so we chose not to use them in the bivariate analysis. **The result of the multivariate analysis was a very high number. Given the difference between the terms / pre-terms (93/3), we do not take into account the result.

## DISCUSSION

We evaluated the virological and immunological responses to ART in HIV pediatric patients who started therapy after the age of 1 year. The patients were born of mothers who were unaware of their HIV status during pregnancy, labor, or breastfeeding. It is noteworthy that the Prevention of Mother-To-Child Transmission (PMTCT) program failed to reach exposed and infected children, and, as such, these children arrived late to our center, since most of them began treatment at the age of around 4.8 years. In addition, all patients underwent at least one hospitalization before being diagnosed with HIV, and opportunities for diagnosis and treatment were missed. The finding of late diagnosis is in line with that in other publications which show different reasons for the delay in diagnosis of children with HIV who have not been enrolled in PMTCT programs^3^ ^-^ ^5^. 

Most of our patients (83.5%) were from Manaus, the capital of the state of Amazonas. Located in northern Brazil, the Amazon state has the same territorial extension as Mongolia and is recognized for its geographical isolation and vulnerability, its immense frontier, the wide diversity in the indigenous groups living in small municipalities or rural communities, and its extreme climate. The area also has the problems of fragile health infrastructure, a lack of well-trained health and laboratory personnel, and a lack of access to CD4^+^ cell count and VL testing, which are centralized in the reference hospital in Manaus^17^ ^,^ ^18^ ^,^ ^19^. The higher concentration of cases in the capital can be explained by the unequal distribution of the population in the interior regions of the state. It may also reflect the difficulty that people in the interior face in getting tested (stigma associated to a positive result and a possible lack of secrecy) and diagnosed and subsequently arriving to our center for treatment. Many publications from Africa and South East Asia have also reported evidence that millions of children living with HIV remain undiagnosed or visit healthcare facilities late in the course of their disease^3^ ^,^ ^4^, either because of gaps in programs outside of PMTCT^3^ or because of reluctance on the part of their parents to have their children tested^3^ ^,^ ^5^. Another reason reported is that services are limited in resource-limited settings and countries that are generally poor^20^ ^-^ ^22^. 

In our study, we found that 96% of the cases of infection were due to vertical transmission and 78.5% of the children were breastfed; however, we were not able to confirm whether these children were infected via breastfeeding or during birth. More than one-third of the patients were orphans (mother/father/both absent). Njom Nlend et al.^23^ reported that being an orphan is associated with treatment failure, but similar to Salou et al.^9^, we did not observe differences in VS between orphans and non-orphans.

Time until ART initiation was the shortest for children aged between 1 and 5 years at HIV diagnosis. The recommendation for universal treatment was mandated in Brazil only in 2017. Until that time, the decision to initiate therapy was based on the VL and immunological status of the patient, and the criteria varied with age. In our study, the youngest age group had the highest initial HIV viral load on arrival when compared to the other two groups, and they were most often classified as CDC categories B and C on arrival. These two reasons could explain why young children received ART soon after arrival at our center. The generally poor virological and immunological state of young children has been described previously^24^ ^-^ ^26^.

Preferred treatment regimens have changed over time, based on research on simplification of regimens, reduction and prevention of adverse effects, and improvement in virological response rates. Initiation of PI-based treatment in all young children is an evidence-based strategy in order to improve suppression rates^26^; however, in our study, improved VS outcomes after 5 years of treatment in patients who started ART with PI regimen were not observed (OR, 0.64; 95% CI, 0.15-2.65).

Young and older children showed a statistically significant increase in CD4^+^ T cell count % over time. However, similar to Cohen et al.^25^ and some other studies^27^ ^,^ ^28^, in our cohort, age at ART initiation was not associated with VS at 5 years, indicating that longer-term immune responses might be independent of the age when ART initiation occurred.

This study highlights the long-term outcomes of VS in pediatric patients in Brazil. To our knowledge, no other Latin American study has assessed VS for such a long time frame^29^ ^-^ ^33^. Regarding other low-and-middle-income countries, as mentioned by Boerma et al.^34^, six studies reported the VS rates after 36 months, but with VL <1000 cps/mL: three studies each for 48 and 60 months. The VS in these studies varied from 70% to 95.5% and the I^2^ described in the meta-analysis was high (>90%), which indicates that the results should be interpreted with caution^34^.

 VS levels were always low for young children at all time points that were assessed, remaining consistently under 37% over the time period analyzed. Previous studies identified factors such as younger age, malnutrition, and advanced stage of disease as causes of attrition among children in ART programs in LMICs, as reported by Boerma et al.^34^. On the other hand, adolescents showed the best VS outcome after 6 months on ART (48.6%). However, they showed a drastic drop to 30%, after 5 years. Lower proportions of VS in adolescents were also a concern in other studies, some showing exceptionally poor virological outcomes in LMICs, thus requiring urgent attention^35^ ^-^ ^37^

VS ranged from approximately 35% to 65% across the analyzed period. Our study and other publications from LMICs presented worse outcomes than high-income countries and were a long way from reaching the 90-90-90 UNAIDS targets^9^ ^,^ ^25^ ^,^ ^27^ ^,^ ^28^ ^,^ ^38^. Studies from high-income countries reported that VS after treatment initiation ranged from 89%^16^ to 92%^24^, but with follow-up studies for no longer than 2 years. A good health care system, adequate clinical situation of patients on arrival, and the possibility of starting treatment earlier would likely contribute to better VS outcomes than those of our pediatric cohort from the state of Amazonas. The results in our analysis did not show any statistical significance in VS over 5 years, which is probably due to our small sample size.

Our study showed that VS was consistently under 65% in the three age groups over time, despite the fact that in Brazil all HIV patients have universal and free access to care and antiretroviral treatment. Two years after ART initiation, VS was stable in children aged 1 to 10 years, and adolescents showed a decline in VS. Boerma et al.^34^ proposed that the scarcity of data after 2 years of follow-up might be an indication of difficulties in retaining children on long-term treatment. However, we did not study linkage, retention, or adherence in the present study.

The limitations of our study include most of the common problems faced when medical records are reviewed (e.g., lack of information, missing data, and heterogeneous records). Data regarding genotyping and adherence were not collected. Many studies involving HIV-infected patients using ART have found a strong association between VS and adherence scale scores^37^ ^,^ ^39^ ^,^ ^40^. The lack of association between patient characteristics and VS might be due to the small sample size in each group. In our bivariate analysis, which explored predictors of suppressed VL at 5 years, we did not consider many “on-ART” variables, such as early virological response after ART initiation, change in caregiver status, social variables, drug adherence and tolerance during the initial period of ART, and comorbidities present on initiation of ART or during ART.

In conclusion, our results offer a broad overview of a long-term registered, vertically-infected HIV population in a middle-income country. This investigation has demonstrated that HIV diagnosis in children and adolescents must be a priority for all health workers, even when children arrive late for relevant care. Despite having mild-to-severe immunosuppression, there is still opportunity for immunological recovery and VS when they start treatment. Free and universal access to care and treatment is not enough to maintain life-long VS levels >90%. Efforts must be made to evaluate long-term VS and understand gaps in the HIV care continuum, and improve comprehensive care for children living with HIV/AIDS. 
